# Fungal biomarker discovery by integration of classifiers

**DOI:** 10.1186/s12864-017-4006-x

**Published:** 2017-08-10

**Authors:** João Pedro Saraiva, Marcus Oswald, Antje Biering, Daniela Röll, Cora Assmann, Tilman Klassert, Markus Blaess, Kristin Czakai, Ralf Claus, Jürgen Löffler, Hortense Slevogt, Rainer König

**Affiliations:** 1 Network Modelling, Leibniz Institute for Natural Product Research and Infection Biology, Hans Knöll Institute (HKI), Beutenbergstraße 11a, Jena, Germany; 2 Center for Sepsis Control and Care (CSCC), Jena University Hospital, Jena, Germany; 3 Septomics Research Centre, Jena University Hospital, Jena, Germany; 4 University Hospital Würzburg, Würzburg, Germany

**Keywords:** Immune response, Systems biology, Fungal pathogens, Microarray, Feature selection

## Abstract

**Background:**

The human immune system is responsible for protecting the host from infection. However, in immunocompromised individuals the risk of infection increases substantially with possible drastic consequences. In extreme, systemic infection can lead to sepsis which is responsible for innumerous deaths worldwide. Amongst its causes are infections by bacteria and fungi. To increase survival, it is mandatory to identify the type of infection rapidly. Discriminating between fungal and bacterial pathogens is key to determine if antifungals or antibiotics should be administered, respectively. For this, in situ experiments have been performed to determine regulation mechanisms of the human immune system to identify biomarkers. However, these studies led to heterogeneous results either due different laboratory settings, pathogen strains, cell types and tissues, as well as the time of sample extraction, to name a few.

**Methods:**

To generate a gene signature capable of discriminating between fungal and bacterial infected samples, we employed Mixed Integer Linear Programming (MILP) based classifiers on several datasets comprised of the above mentioned pathogens.

**Results:**

When combining the classifiers by a joint optimization we could increase the consistency of the biomarker gene list independently of the experimental setup. An increase in pairwise overlap (the number of genes that overlap in each cross-validation) of 43% was obtained by this approach when compared to that of single classifiers. The refined gene list was composed of 19 genes and ranked according to consistency in expression (up- or down-regulated) and most of them were linked either directly or indirectly to the ERK-MAPK signalling pathway, which has been shown to play a key role in the immune response to infection. Testing of the identified 12 genes on an unseen dataset yielded an average accuracy of 83%.

**Conclusions:**

In conclusion, our method allowed the combination of independent classifiers and increased consistency and reliability of the generated gene signatures.

**Electronic supplementary material:**

The online version of this article (doi:10.1186/s12864-017-4006-x) contains supplementary material, which is available to authorized users.

## Background

The human body is protected by an immune system capable of tackling most of the microorganisms it encounters. Severity of infections is dependent, not only on the type of pathogen it encounters, but also on the rapid and effective response of the immune response as well as the site of infection [1]. If the infectious agent has established itself and evaded the immune system, it may cause sepsis when it disseminates (systemic infection) throughout the body with possible deadly consequences to the human host [2]. Sepsis is a life threatening disease caused by systemic infection followed by an uncontrolled immune response and organ dysfunction. Therapy consists of clearance of the infection, administration of antibiotics and clinical supportive measures.

Most often sepsis is caused by bacterial infection. In turn, fungal opportunistic pathogens such as *C. albicans*, which mainly causes infections of the mucosa [2], may, in immunocompromised individuals or when the epithelial barrier is not effective, spread into the body through the blood system. Additionally, fungal infections may arise during hospital admission by use of invasive monitoring techniques such as invasive blood pressure monitoring procedures or during arterial catheterization [3]. Hence, systemic infection can arise from fungal or bacterial contamination of the blood.

Importantly, it is essential to quickly and precisely determine the cause of the disease in order to employ the appropriate antibacterial or antifungal treatment for clearing the infection. Employment of the wrong therapy in infected individuals clearly has no impact and may even promote increased infection due to effecting of the normal gut flora allowing for proliferation of fungal opportunistic pathogens such as *Candida albicans* [4]. Indeed, unable to provide accurate and early diagnosis places patients at much higher risk to die [5, 6].

Currently, blood cultures are the “gold standard” for the identification of pathogens in the blood. However, this approach can take several days to identify the infectious agent [7]. A quicker way of diagnosis would be to examine the direct host response of the infection in the blood. Despite all efforts, no clear generic host gene signature for distinguishing fungal from bacterial infections exist to date. Identifying robust biomarkers to discriminate fungal from bacterial infection is difficult as the complexity of the immune response has many variables such as the composition and ratio of immune cell types, site of infection, host immune status, stage of infection, age of the patient and concomitant infections. The use of transcriptomics in biomarker discovery is increasingly promising in infection biology. Dix and co-workers employed a classification based approach identifying genes capable of distinguishing infected from non-infected and fungal from bacterial infected human blood [8]. Other transcriptomics studies also investigated the human immune response to fungal pathogens but yielded different gene signatures [9–12].

These approaches drawback in a lack of consistency of the predicted gene signatures across studies. Laboratory settings, culture conditions, different compositions of cell types, time of sample extraction, methods of sequencing and even the same experimental setups performed at different days can lead to different results. It is vital that methods are developed to identify consistent biomarker gene signatures which are rather independent of these parameters. Consistency, in this case, refers to similar gene signatures, irrespective of the above mentioned variables, for a specific disease or infection.

To tackle this issue, we employed a constrained based method based on Mixed Integer Linear Programming (MILP). MILPs have been used for the optimization of cell-network arrangements in order to discover patterns in pathways which are distinctively expressed [13], in the inference of gene regulation [14] and in the identification of gene signatures capable of distinguishing infected from non-infected samples [15]. A major advantage of applying our method resides on the reduction of the search space on which the optimization problem is performed by the imposition of constraints. In the present study, MILPs were employed to combine classification problems across several datasets of fungal and bacterial infections. Two independent optimization problems were combined by constraining them to use the exact same set of features, thus improving the consistency of the predicted biomarkers, irrespective of the experimental conditions and confounders. Rather than focusing on performance enhancements, our main goal was to identify a set of genes that could distinguish fungal from bacterial infected samples in a consistent manner.

## Methods

### Dataset assembly

Normalized gene expression data from three datasets was downloaded from Gene Expression Omnibus (GEO, http://www.ncbi.nlm.nih.gov/geo/). The first dataset we downloaded (accession number GSE42606) was from a study by Smeekens and co-workers [11]. In summary, it consisted of data from Peripheral Blood Mononuclear Cells (PBMCs) from healthy donors challenged with heat-killed *C. albicans*, *M. tuberculosis* and LPS (lipopolysaccharides) from *E. coli*. A total of 73 samples (24 fungal and 49 bacterial, extracted 4 h post-infection) were collected. Further, gene expression data of a study carried out by Czakai and colleagues [12] (GSE69723) was generated by challenging healthy blood-derived human dendritic cells (DCs) with thimerosal treated *C. albicans* SC5314 (MOI of 1), *A. fumigatus* ATCC 46645 (MOI of 1) and LPS (from *E. coli*) (1 μg/ml). Four samples of each infection type were collected (4 h after infection). Dix and co-workers [8] performed a study where healthy human anticoagulated blood was challenged with either thimerosal treated *A. fumigatus* ATCC 46645, *C. albicans* SC5314 (each at 1 × 10^6^/ml), *S. aureus* ATCC25923 (1 × 10^6^/ml), or *E. coli* ATCC25922 (each at 4 × 10^3^/ml) for 4 and 8 h (GSE65088). Samples were grouped into bacterial (*n* = 20) and fungal (*n* = 16) infection. A study performed by Klassert and co-workers [16] consisted of data from healthy human blood-derived monocytes challenged with either heat-killed *C. albicans* SC5314 yeast (MOI of 1), *A. fumigatus* AF293 (MOI of 1) or *E. coli* serotype O18:K1:H7 (MOI of 10). For our study, we used data of a total of *n* = 27 samples which were extracted 3 and 6 h post-infection (*n* = 9 bacterial and *n* = 18 fungal). We used transcriptomic data generated by Saraiva and co-workers [15] from healthy human isolated PBMCs which were challenged with either heat-killed *C. albicans* MYA-3573 yeast (MOI of 2) or LPS (10 ηg/ml) from *E. coli* 0111: B4 (InvivoGen). Four samples were extracted 4 h post-infection of each stimulus. RNA was extracted using RNAEasy Kit Qiagen and quantity and quality of the total RNA was analyzed using a Nanodrop ND − 1000 spectrophotometer (Thermo Fischer Scientific, USA) and a Tape Station 2200 (Agilent Technologies, USA). Normalization of the dataset from Saraiva and co-workers was achieved by applying the functions “lumiN” and method “vsn” of the “lumi” R package [17]. All downloaded datasets were already normalized so no further actions in this direction were performed. To eliminate possible duplicate gene entries, mean expression values were calculated using the “avereps” function of the “limma” R package [18]. To focus on high expressed and high variant genes, genes were removed from the data that presented a variance and standard deviation below 40%. Finally, z-scores were calculated for each gene. This procedure was done for all datasets separately. We next intersected the gene lists from each dataset and obtained a final list of 1516 genes to be used for feature selection and classification.

### Support vector machine implementation

The MILP implementation of the SVM was realized as follows: The objective function was defined as the maximization of the margin of the SVM as seen in Eq. 1,

1 $$ {obj}_{classifier}=\mathit{\max}\left({t}_1-{t}_2\right), $$

with *t*_*1*_ and *t*_*2*_ are the margins of class 1 and class 2 to the separating hyperplane, respectively. The objective was subjected to the following constraints. Equations 2 and 3,

2 $$ \sum \limits_{i=1}^{nGenes}{n}_i{g}_{ij}\ge {t}_1-{My}_j{\forall}_j\in {C}_1, $$

3 $$ \sum \limits_{i=1}^{nGenes}{n}_i{g}_{ij}\le {t}_2+{My}_j{\forall}_j\in {C}_2, $$

define the constraints applied to the classifier, for class 1 (C_1_, fungal) and class 2 (C_2,_ bacterial), respectively. The scalar product of the gene expression of sample *j* with the weight *n* (for all genes *i* ϵ {1, …, nGenes}) assigned them to a specific side of the margin but only for samples whose variables *y*_*j*_ ϵ {0,1} were equal to 1. If this scalar product was less or equal than *t*_*2*_ than the samples were classified as bacterial and if greater or equal to *t*_*1*_, classified as fungal. M was a large constant (“big M”) that was set to allow exceptions if *y*_*j*_ equaled 1. Equation 4,

4 $$ \sum \limits_{j=1}^{nSamples}{y}_j\le k $$

constrained the number of allowed misclassifications *k* during the training (with nSamples training samples) of the classifier. k was set to 10% of the total number of samples |*S*|. In order to ensure that only genes *i* whose corresponding variables x_i_ ϵ {0,1} equaled to 1 were used for classification, constraints of eqs. 5 and 6 were established,

5 $$ {n}_i\le {x}_i\forall i\in G $$

6 $$ -{n}_i\le {x}_i\forall i\in G. $$

The number of features (genes) which should be determined was constrained by eq. 7, in our present study *l* was set to 30,

7 $$ \sum \limits_{i=1}^{nSamples}{x}_i\le l. $$

*x* and *y* were defined as binary variables which belong to the set of genes *G* and samples *S* by:

$$ {x}_i\in \left\{0,1\right\}\forall i\in G $$

$$ {y}_j\in \left\{0,1\right\}\forall j\in S $$

**Fig. 1:**
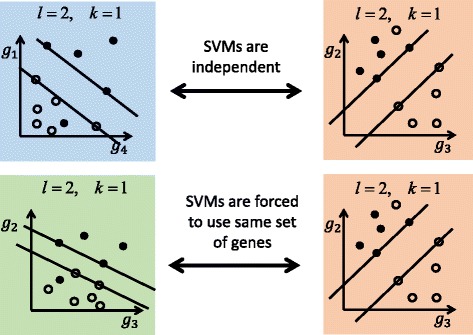
The upper two SVMs maximize the margin independently. The lower two SVMs maximize the sum of the two margins, but are constrained to use the same set of genes for features. Obviously the margins cannot increase but note that the overall SVM efficiencies were as good as before after applying these conditions.

To note, applying these sets of constraints generated a MILP problem and not an ordinary Linear Programming (LP) problem. Selection of consistent genes across all datasets required the combination of two independent MILPs. Each independent classifier was established by applying all previously defined equations. Next, the problems were connected by a combined objective function, Eq. 8,

8 $$ {obj}_{combined}={obj}_{classifier1}+{obj}_{classifier2} $$

adding up the objective functions of each classifier. Using identical *x* variables in both classifiers ensured that they use the same set of features, possibly leading to a decrease in performance of a single classifier (Fig. 1).

### Machine learning implementation and statistical analysis

Support Vector Machines are, in principle, designed for balanced classes, although sub-optimal results can still be ascertained having unbalanced classes in the datasets. Indeed, training of SVM classifiers on imbalanced datasets will often generate models biased towards the class with the highest number of samples [19]. Two prevent this effect we employed stratification in each classification problem. Cross-validation was employed in which 2/3 of the samples from each class (fungal and bacterial) were randomly chosen for training in which we allowed 10% of the samples to be misclassified. Random samples of the majority class were selected that amounted to the number of samples in the minority class. The remaining samples were used for validation in order to determine classifier performance. This procedure was repeated 100 times generating 100 lists of selected genes used as features of the SVMs. For comparability, we constrained the number of selected genes to *n* = 30. Performance was assessed by accuracy of the classification on the validation sets. Regarding the cases in which two classifiers were combined, the average of the performances was calculated. To compare the performances of single classifiers with the performances of combined classifiers, we calculated the overall average from the single classifiers and combined classifiers, respectively. Consistency of selected genes was calculated for each pair of lists of selected genes by calculating the pairwise overlap (POL) between the 100 gene lists generated during classification of the two datasets in question. The mean POL and standard deviations (1б) were calculated from the list of POL. The final list of intersecting genes was obtained by taking the union of genes from each classifier that were selected in at least 40% of the cross-validation runs.

To evaluate the overall quality of the generated models we determined several performance metrics such as sensitivity, specificity, positive predictive value (PPV), negative predictive value (NPV) and accuracy. As benchmark, the average across all single classifiers, of each of these performance measures was calculated. For the combined classifiers the average was taken for all combinations of classifiers. Differential gene expression was calculated using Student’s t-tests and multiple testing correction was performed by the Benjamini-Hochberg method [20]. Genes were considered to be differentially expressed if their adjusted *p*-value was below 0.05. All statistical analyses were performed using R software (http://www.r-project.org/) and packages from Bioconductor [21]. MILP implementation was also performed in R using the Gurobi interface library and solved with the Gurobi solver (version 6.5.1, www.gurobi.com).

## Results

### Rationale and workflow

We intended to obtain a consistent gene signature to distinguish fungal from bacterial infections. We implemented a classification problem using linear Support Vector Machines (SVMs) to select the most discriminate features. Two independent optimization problems were defined, each based on a separate dataset. Each problem consisted of optimizing the margin of an SVM for each classifier based on two different datasets. Now, within the same optimization problem, we enforced the classifiers to use the same feature sets. This led to solve an optimization problem for pairs of classifiers. The feature selection dependency limited the search space and features (genes) were only selected if they were discriminative for both classification problems (see Fig. 1). In this manner, collaborative selection of features was enforced which was intended to improve consistency of gene selection.

**Fig. 2:**
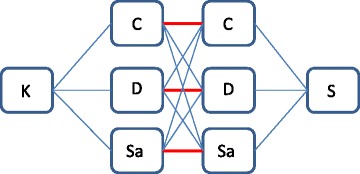
Benchmark results were compared to the combined approach by intersecting the gene lists of each combination which contained one of the datasets (here exemplarily shown for Smeekens) with each combination containing the other dataset (here: Klassert). We did not consider the intersections, highlighted in red, in which one of the datasets occurred on both “sides” of the combination (e.g. combinations Klassert & Czakai versus Smeekens & Czakai; or Dix & Klassert versus Smeekens & Dix)

For determining classification performance and to get an estimate of variance of the selected features, each pair of classifiers was run within a cross-validation procedure, randomly selecting different sets of samples used for training and validation. Pairwise overlap (POL) of each “combined” classifier was calculated with another “combined” classifier composed of different datasets to those in the first. For instance, the gene overlap of the classifier which was composed of Smeekens and Dix was only calculated with selected genes from classifiers based on two sets of Klassert and Czakai and Klassert and Saraiva (see Fig. 2). This procedure was performed for all possible combinations of the different runs of the cross-validations yielding the averaged pairwise overlap. As a benchmark, the averaged POL using the single classifiers of each of the datasets was used. To estimate if the classification performance decreased if constraining them to use the same features, the performances of the combined classifiers were compared to the performances of the single classifiers. Finally, a ranked list of genes which were most often selected by the combined classifiers was assembled and top ranking genes were selected as biomarkers if they were consistently differentially expressed in a majority of the analyzed datasets.

### Combining classifiers improves consistency

For each dataset the classifiers ran on 100 randomly selected different training sets. Each run yielded a list of 30 genes which best discriminated between fungal and bacterial infection. The POL of the 100 gene lists from each single classifier returned an average value of 1.09 (1б = 0.35) (Additional file 1: Table S1 for the results of each dataset POL and average). Our main goal was to obtain a *consistent* gene signature for distinguishing fungal from bacterial infections, independent of the experimental setup. For this, we combined two classifiers from different datasets. We calculated the POL for these combined classifiers as described above and obtained an average POL of 1.57 (1б = 0.46). It should be noted that as stated in the methods section, the POL was only calculated for distinct datasets. Our combined approach improved the consistency of our gene signature by 43% when compared to that of single classifiers which demonstrates the benefits of our method. This difference was significant (*P* = 2.2E-16 using a two-sided Kolmogorov-Smirnov test). Next, we combined the gene lists from all single classifiers and obtained a total of 72 genes (Additional file 1: Table S2). To note, we only intersected genes that were selected in at least 40% of the runs. We performed the same procedure for the combined classifiers and obtained a gene list composed of 88 genes (Additional file 1: Table S2). Comparing the gene lists from each approach showed that out of the 72 single classifier genes, 46 also were selected by the combined approach which corresponds to 64%. We performed gene set enrichment analysis of the resulting lists (intersection, and both classifiers approach specific) in order to obtain a functional overview of each of them using The Database for Annotation, Visualization and Integrated Discovery (DAVID, version 6.7, https://david.ncifcrf.gov/home.jsp) [22]. Gene Ontology terms such as immune response, purine nucleotide metabolic process and cell death and regulation of alpha-beta T cell activation were enriched using the combined classifier specific gene list. Single classifier specific genes were enriched in negative regulation of catalytic activity. We were interested which genes were consistently selected across all runs. Five genes were used in more than 70 runs for the combined classifiers, but only three single classifier specific genes were used in more than 70 runs. For the latter, we got the genes T cell activation RhoGTPase Activating Protein (TAGAP), ArfGAP with GTPase Domain (AGAP3) and C-C Motif Chemokine Ligand 8 (CCL8). The first gene, as suggested by its description is involved in the activation of T cells. This gene is also a target of vitamin D [23], which has been shown to modulate inflammation and regulation of calprotectin expression which, in turn, influences neutrophil activity linked to oral candidiasis [24]. AGAP3 is a GTPase activating protein that may be involved in the degradation of expanded polyglutamine proteins via the ubiquitin-proteasome pathway. How this gene is related to fungal infections still remains elusive. Lastly, CCL8 is a cytokine that displays chemotactic activity for several immune cells (e.g. monocytes and lymphocytes) recruiting them to sites of inflammation and has been shown to be a ligand for several C-C motif receptors (CCRs) such as CCR1 [25, 26]. Considering the combined classifier specific genes, 5 were selected in more than 70 runs: C14orf159, SLC16A3, SLC7A7, KLF4 and EMP1. Solute Carrier Family 16 member 3 (SLC16A3) encodes a protein involved in the transport of monocarboxylates such as lactate, pyruvate and branch-chained amino acids derived from leucine, valine and isoleucine. Glycolysis is essential in the activation of the innate immune response. Enhanced glycolysis leads to increased lactate production. Extracellular transport of these products is performed by SLC16A3. Studies have shown that up regulation of SLC16A3 maintains a high rate of glycolysis and, subsequently, the immune response [27]. Similar to the latter, SLC7A7 is involved in the sodium-independent transport of bibasic amino acids and sodium dependent neutral amino acids. Studies have suggested a role in nitric oxide synthesis in human umbilical endothelial cells (HUVECs) through L-arginine transport [28]. Arginine has been shown to play an important role in the differentiation of monocytes into macrophages [29]. Kruppel-Like Factor 4 (KLF4) encodes a transcription factor involved in both inhibition as well as activation of target genes. Studies have shown that it modulates interleukin 6 (IL6) release in human dendritic cells (DCs) following fungal infection [12]. Epithelial membrane protein 1 (EMP1) encodes a protein involved in many processes related to cell proliferation and differentiation. This gene has been linked to several cancer types [30–32], however, to our knowledge, not to fungal infections so far. The mere analysis of the top classifier specific genes already shows the increase in biological information extraction from the combined classifier approach when compared to the single classifiers. Although 46 genes were selected by both single and combined classifiers, the average number of runs the genes were selected was higher using the combined approach. Common single and combined classifier genes were, on average, selected in 55 and 60 runs, respectively. These results already demonstrate a clear improvement in consistency when employing our combined classifiers.

Next, we calculated differential gene expression of the selected genes for each dataset. We discarded genes which were not differentially expressed in at least four (out of five) datasets. From our biomarker list, 19 met this criterion and, out of these, 12 were constantly upregulated in all datasets. The respective gene list and differential expression are shown in Table 1, whilst their adjusted *p*-values are shown in Additional file 1: Table S3 in the Supplementary data. Genes that were up-regulated in all datasets are ranked as the highest followed by genes that were down-regulated in all datasets and lastly by the remaining non-homogenously directional genes. Within each group they are further ranked by highest average number of times they are selected in each combined classifier. In the following, we regarded this gene list as our refined biomarker list and discuss its functional relevance below (section 3.4). As seen in Table 2, eight combined classifier specific genes were present in the refined biomarker gene signature and no single classifier specific genes. The refined biomarker gene list contained 11 genes that were selected using both approaches.

**Table 1:** Refined list of biomarker genes and their regulation across the investigated datasets

Gene	Dix	Smeekens	Saraiva	Klassert	Czakai	Average N° runs
HMOX1	1**	1	1	1	1	71
CCR1	1	1	1	1	1	61
GLA	1	1	1	1	1	48
TNFSF14	1	1	1	1	1	60
TBC1D7	1	1	1	1	1	65
SPRY2	1	1	1	1	1	63
EGR2	1	1	1	1	1	60
BCAR3	1	1	1	1	1	59
PAPSS1	1	1	1	1	1	58
RRAGD	1	1	1	1	1	55
DHRS9	1	1	1	1	1	54
SDSL	1	1	1	1	1	53
RNF144B	−1	−1	−1	−1	−1	67
ADA	−1	−1	-1	-1	-1	56
SCARB2	1	1	1	1	-1	64
SOWAHC	1	1	1	1	-1	55
BLVRA	-1	1	-1	-1	-1	64
EDN1	1	1	1	1	-1	97
TNFSF15	1	1	1	1	-1	53

**1: up-regulated, −1: down-regulated, in fungal versus bacterial infected immune cells

**Table 2:** Single and combined classifier gene lists

Intersection of combined and single classifiers	Combined only
*HMOX1*	*GLA*
*CCR1*	*EGR2*
*TNFSF14*	*BCAR3*
*TBC1D7*	*SDSL*
*SPRY2*	*RNF144B*
*PAPSS1*	*ADA*
*RRAGD*	*BLVRA*
*DHRS9*	*TNFSF15*
*SCARB2*	
*SOWAHC*	
*EDN1*	

### The classification performances of the combined classifiers were comparable to the single classifiers

The combined classifiers had a mean value of 0.96 for sensitivity, 0.97 for specificity, 0.97 for positive predictive value (PPV), 0.96 for negative predictive value (NPV) and 0.96 for accuracy. Single classifiers presented practically identical values (maximum difference of 1 %) demonstrating that our method did not decrease performance. The full performance results can be found in Additional file 1: Tables S4 and S5 in the Supplementary material.

### Test data of monocytes challenged with LPS and *A. fumigatus*

To get an estimate of the generalizability of our identified biomarker list, we applied the biomarker list to a new, unseen dataset. In order to determine if the sample is infected with a fungal or bacterial pathogen, we used only the expression values of the 12 genes from the refined biomarker list that were up-regulated in all datasets. Classification was performed by employing random forest classifiers (available through the “caret” R package, version 6.0–71), trained on each of our datasets (Smeekens, Dix, Czakai and Klassert), except the Saraiva dataset which was too small for getting reliably trained classifiers (4 fungal and 4 bacterial datasets). Measuring the performance of our classifiers (i.e. capacity to distinguish fungal from bacterial infected samples) required that the testing samples, the ones we wish to classify, be independent from the training samples used to build the classifiers. For this we used unseen/new microarray data (from ArrayExpress, www.ebi.ac.uk/arrayexpress, E-MEXP-1103) consisting of 6 samples of human monocytes challenged with LPS and 5 samples challenged with *A. fumigatus*. Samples were extracted 6 h post-infection. Table 3 shows all the performance results. Strikingly, analysing classification performance using the new data, all our models classified the fungal samples with more than 73% accuracy, yielding an average of 87%. Average sensitivities and specificities yielded an average of 79% and 100%, respectively. All misclassified samples belonged to the bacterial class.

**Table 3:** Performance using our identified gene signature on unseen data

	Dix	Klassert	Smeekens	Czakai	Average
Sensitivity	0.71	0.83	1	0.63	0.79
Specificity	1	1	1	1	1
PPV	1	1	1	1	1
NPV	0.67	0.83	1	0.5	0.75
Accuracy	0.82	0.91	1	0.73	0.87

### Functional roles of the refined biomarker genes

In the following, we will discuss the top ranking genes from our biomarker gene list that were consistently differentially expressed (i.e. from the refined biomarker list) and which were up-regulated in host cells challenged with fungal infections compared to bacterial infections. The Breast Cancer Antigen-estrogen Resistance 3 (BCAR3) is a gene related to estrogen resistance. Overexpression of the latter has been shown to activate CDC42 [33] which, in turn, participates in signalling pathways related to cellular functions such as cell morphology, endocytosis, cell cycle progression and T cell activation. In addition, CDC42 induces phagocytosis in macrophages via FcγR [34]. A study using human polymorphonuclear leukocyte (PMNs) showed that BCAR3 was down-regulated by gliotoxin, a mycotoxin produced by *A. fumigatus*, which further enhances the importance of this gene in the host immune response during fungal infection [35]. Early Growth Response 2 (EGR2) is a transcription factor closely related to HOXA4 gene and has been shown to be up-regulated during *C. albicans* infection in bone marrow derived macrophages [36]. Macrophage and dendritic cell inflammatory response restrictions have also been associated with Egr2 and Egr3 when induced by Dectin − 1 [37]. Studies have shown that Dectin − 1, a well-known receptor for recognition of fungal β − 1,3-glucans triggers NFAT activation which, in turn, regulates the induction of EGR transcription factors [38]. The NFAT transcription factor family has roles in processes such as thymocyte development, T cell differentiation and activation. Early growth response transcription factors have been shown to mediate production of reactive oxygen species (ROS) and TNFα which, in turn, have been linked to immune responses [39]. Galactosidase A (GLA) is a gene involved in recycling processes within the cells, converting mellibiose into galactose and glucose. A study by Bhavan and colleagues [40] demonstrated that *C. albicans* required mellibiose but was incapable of assimilating it. This suggested that the human host may get triggered to activate genes in this process facilitating carbon uptake of the fungal pathogen. Heme oxygenase 1 (HMOX1) is involved in the cleavage of the heme ring at the α-methene bridge to form biliverdin. Excess free heme has been demonstrated to sensitize cells to undergo apoptosis and heme oxygenease 1 protects cells from this process [41]. It has also been shown that HMOX1 expression is up-regulated during events such as oxidative stress or in the presence of inflammatory cytokines (Poss & Tonegawa, 1997). The immune system, upon recognition of infection, induce the production of inflammatory cytokines and chemokines. Both bacteria and fungi infected cells induce the expression of heme oxygenase 1, however, our results suggest a more protective effect of human immune cells during fungal infection. *C. albicans* also encodes HMOX1 which is induced by hemoglobin [42]. Studies have shown that *C. albicans* increases its adhesion to host cells in the presence of hemoglobin and, subsequently, infection dissemination. The same study demonstrated that hemoglobin induced *C. albicans* HMOX1 enzyme activity produced biliverdin which lead to the hypothesis that this gene provided the fungal pathogen with a growth nutritional advantage in the human host. Biliverdin was also suggested to have protective effects on *C. albicans* killing by phagocytosis [43]. This leads us to the next gene which is consistently differentially expressed in our list of biomarker genes, Biliverdin Reductase A (BLVRA). In this case, BLVRA is down-regulated in all datasets except that of Smeeken’s. Amongst its functions is the conversion of biliverdin to bilirubin which has a potent antioxidant activity. The latter is converted back into biliverdin via ROS allowing their neutralization. Apart from its reductase activity, this gene has also been shown to activate cellular signalling pathways such as the MAPK signalling cascade. This suggests a complex regulation of the production of ROS and several cell signalling pathways that should be further investigated [44]. Sulfate adenyltransferase 1 (PAPSS1) is involved in sulfate activation. Sulfite has antimicrobial and antioxidant properties and has been shown to be toxic for *C. albicans* [45]. The upregulation of this gene could indicate a possible defence mechanism of the human host to fungal pathogens. Ras-related GTP binding D (RRAGD) is a gene involved in activation of the mammalian target of t﻿he rapamycin (mTOR) signalling cascade by amino acids [46–48]. Studies have shown that mTOR plays a fundamental role in the protection of epithelial cells during fungal infections [49] as well as in the induction of monocytes via trained immunity [50]. Serine Dehydratase like (SDSL) gene encodes a serine protease. These proteases are involved in several processes which include blood coagulation, apoptosis and inflammation. The regulation of proteases has been shown to prevent self-induced damage [51]. However, no information is available which link the human host immune response and the expression of SDSL during fungal infections. Sprouty RTK Signalling Antagonist 2 (SPRY2) acts as a negative regulator of the MAPK-ERK signalling pathway [49]. Studies have shown that this gene was up-regulated in mouse peritoneal fibroblasts challenged with *C. albicans* [52]. C-C Chemokine Receptor 1 (CCR1) encodes a member of the chemokine receptor family. Chemokines and their respective receptors have important roles in the recruitment of effector immune cells to sites of infection [53]. CCR1 knockout mice presented higher mortality, compared to the wild type, when infected with *A. fumigatus* which suggests a role in the control of fungal infections [54]. This is not surprising since chemokines and their receptors have been shown to play central roles in the recruitment of immune cells to sites of fungal infection, regulation of cytokine production and antigen presentation [26]. Nevertheless, in other studies CCR1 knockout mice show, in the late phase of invasive candidiasis, impaired accumulation of neutrophils in the kidney associated with improved renal function and survival without impact on tissue fungal burden [25]. In line with this, late onset of antagonistic inhibition of CCR1 in chronic fungal asthma caused by *A. fumigatus* attenuated late disease features such as peribronchial inflammation [55]. These results suggest that CCR1 knockout in the host at early stages of fungal infection is detrimental whilst at later stages is advantageous. Finally, Cell Migration-Inducing Protein 23 (TBC1D7) is a negative regulator of the mTORC1 signalling cascade by acting as a GAP (GTPase activating) protein for Ras homolog enriched in brain (RHEB). Since RHEB has been identified as an activator of the MAPK signalling cascade regulating cellular processes such as cell growth and proliferation [47], its inactivation via TBC1D7 may affect the activity of the MAPK signalling pathway.

Summarizing, we identified a gene signature being highly relevant in the response of human immune cells due to fungal infection when compared to bacteria, in particular, comprising genes of the MAPK kinase activation pathway.

## Discussion

In the present study we aimed identify biomarker gene lists for distinguishing fungal from bacterial infections across several datasets. Our combined classifier approach produced a consistent list composed of 75 genes. Following differential expression analysis and imposing that genes should be differentially expressed in at least 4 datasets, decreased the list to 19 genes of which 12 were consistently up-regulated in all datasets which we selected as the refined biomarker gene set. We employed Mixed Integer Linear Programming to extend the classification problem. MILP, unlike ordinary LP, allows the modelling of discrete variables and constraints such as defining the number of misclassifications allowed during classification. However, as in other classification approaches, MILPs also have disadvantages such as the theoretically long running time required to obtain the perfect solution. Limiting the amount of time expended to obtain the optimized solution by use of solvers such as gurobi decreases the latter, while still presenting good solutions. To note, a previous study which we presented on a conference (see [15]) has demonstrated an initial implementation of our approach in the identification of consistent gene signatures capable of discriminating between infected and healthy samples. This was a more straight forward application when compared to the discrimination of the kind of infection (fungal versus bacterial).

Gene signatures, in general, lack consistency across studies due to differing experimental procedures, pathogen strains, laboratory settings and even between samples processed in equal conditions but collected at different times. With our approach, we were able to identify a set of genes capable of distinguishing between fungal and bacterial infected samples with an average accuracy of 96%. The pairwise overlap (number of genes consistently selected across runs) was 43% higher than that of the single classifiers which shows an immediate improvement in feature identification without introducing prior knowledge into the feature selection and classification problem. Our method showed no decrease in performance when compared to single classifiers. From the genes consistently selected by single classifiers, 64% were also identified by our combined classifier approach which demonstrates that combining classifiers does not result in a completely different gene list. We tested whether our 12 consistently selected, differentially expressed and up-regulated genes were capable of distinguishing fungal infected from bacterial infected samples in a dataset not used during the training and feature selection. The gene list was tested on new data, yielding an average accuracy of 87%. Interestingly, misclassification of samples only occurred for bacterial infected samples which is shown by a perfect score in terms of specificity. This may have an advantage for clinical transfer as in particular the comparably less often occurring fungal systemic infections need to be precisely identified during sepsis.

Several of the consistently differentially expressed genes selected by our approach show a strong link to the MAPK signalling pathway either directly or indirectly. The MAPK signalling cascade is highly conserved across species and its role in the regulation of gene expression during infection or stress-related events has been well described [56]. Fungal cell wall biogenesis, morphogenesis and environmental adaptation has also been shown to be influenced by the MAPK signalling cascade in *A. flavus* [57, 58]. Despite our main goal being the host response towards fungal infection, how both host and pathogen MAPK signalling cascades are regulated during infection should be addressed in future studies. Additionally, our identified gene signature should be empirically verified by studies on gene expression in animal models and in samples from septic patients. All donors were healthy and the immune cells were isolated from their blood and cultivated in vitro in medium containing antibiotics. Pathogens were grown, killed and used to challenge the immune cells in a very controlled standardized procedure, independent from the healthy donors. It is known that there is a strong interplay between the gut microbiome of the host and its immune system. As future work, it might be intriguing to shape out differences in immune response which may be due to this interplay. Our method allows the integration of additional information such as protein-protein interaction networks, which could provide additional insight on how these genes are connected. Our method also allows the combination of multiple classifiers with the respective increase in computational running time. In our present study, no optimization on the number of features for classification was performed. No significant difference, in terms of performance and pairwise overlaps, was obtained when using 20 rather than 30 genes for classification in a previous study [15] which suggested that, while not optimal, a different selection of the constrained number of features would not lead to much different results.

## Conclusions

In summary, employing our approach combining classifiers constrained to base on the same set of selected features led to a consistent biomarker gene signature across the investigated datasets. It was robust enough to obtain good classification results when tested on an unseen dataset and the obtained biomarker genes are functionally plausible in demonstrating the different regulatory response mechanisms to fungal when compared to bacterial infections.

## Additional file

Additional file 1: Table S1. Lists of pairwise overlaps. **Table S2.** – List of biomarker genes from each type of classifier. **Table S3.** List of genes selected from the combined approach and their respective adjusted p-values (<0.05 was regarded to be significant). **Table S4.** Single Classifier Performances. **Table S5.** Combined Classifier Performances. (DOCX 24 kb)

## Abbreviations

AGAP3: ArfGAP with GTPase Domain
BCAR3: Breast Cancer Antigen-estrogen Resistance 3
BLVRA: Biliverdin Reductase A
CCL8: C-C Motif Chemokine Ligand 8
CCR1: C-C Chemokine Receptor 1
CCRs: C-C motif receptors
CDC42: Cell division control protein 42
DCs: Dendritic cells
EGR2: Early Growth Response 2
EGR3: Early Growth Response 3
EMP1: Epithelial membrane protein 1
GAP: GTPase activating protein
GLA: Galactosidase A
HMOX1: Heme oxygenase 1
HOXA4: Homeobox A4
HUVECs: Human umbilical endothelial cells
IL6: Interleukin 6
KLF4: Kruppel-Like Factor 4
LP: Linear Programming
LPS: Lipopolysaccharides
MAPK: Mitogen-activated Protein kinase
MILP: Mixed Integer Linear Programming
MOI: Multiplicity of Infection
mTOR: Mammalian target of rapamycin
NFAT: Nuclear factor of activated T-cells
NPV: Negative predictive value
PAPSS1: Sulfate adenyltransferase 1
PBMCs: Peripheral Blood Mononuclear Cells
PMNs: Human polymorphonuclear leukocyte
POL: Pairwise overlap
PPV: Positive predictive value
RHEB: Ras homolog enriched in brain
ROS: Reactive oxygen species
RRAGD: Ras-related GTP binding D
SDSL: Serine Dehydratase like
SLC16A3: Solute Carrier Family 16 member 3
SPRY2: Sprouty RTK Signalling Antagonist 2
SVM: Support Vector Machine
TAGAP: T cell activation RhoGTPase Activating Protein
TBC1D7: Cell Migration-Inducing Protein 23
TNFα: Tumor necrosis factor alpha
