# Supplementary material for: Fungal biomarker discovery by integration of classifiers
Source: BMC Genomics. 2017 Aug 10;18:601. doi: 10.1186/s12864-017-4006-x (PMC5553868; doi:10.1186/s12864-017-4006-x)
Supplement: Additional file 1: Table S1. — Lists of pairwise overlaps. Table S2. – List of biomarker genes from each type of classifier. Table S3. List of genes selected from the combined approach and their respective adjusted p-values (<0.05 was regarded to be significant). Table S4. Single Classifier Performances. Table S5. Combined Classifier Performances. (DOCX 24 kb) [file 12864_2017_4006_MOESM1_ESM.docx]

**Fungal biomarker discovery by integration of classifiers**

João Pedro Saraiva ^1,2^, Marcus Oswald ^1,2^, Antje Biering ^1,2^, Daniela Röll^1,2^, Cora Assmann ^3^, Tilman Klassert ^3^, Markus Blaess^2^, Kristin Czakai ^5^, Ralf Claus^2^, Jürgen Löffler ^4^, Hortense Slevogt ^3^, Rainer König ^1,2,*^

*corresponding author

*^1^Network Modelling, Leibniz Institute for Natural Product Research and Infection Biology, Hans Knöll Institute (HKI), Beutenbergstraße 11a , Jena, Germany, ^2^ Centre for Sepsis Control and Care (CSCC), Jena University Hospital, Jena, Germany (e-mail: rainer.koenig@uni-jena.de), ^3^ Septomics Research Centre, Jena University Hospital, Jena, Germany, ^4^University Hospital Würzburg, Würzburg, Germany*

**Supplementary material**

Table S1 Pairwise overlaps

| Single classifiers | | Combined classifiers | |
| --- | --- | --- | --- |
| Pair | POL | Pair | POL |
| C_D | 0.57 | D_C_Sa_K | 1.53 |
| C_K | 1.39 | D_C_S_K | 1.13 |
| C_Sa | 0.76 | D_C_S_Sa | 1.27 |
| C_S | 0.96 | D_K_Sa_C | 2.14 |
| D_K | 0.82 | D_K_S_C | 1.82 |
| D_Sa | 1.53 | D_K_S_Sa | 1.32 |
| D_S | 1.02 | D_Sa_K_C | 1.01 |
| K_Sa | 1.07 | D_Sa_S_C | 2.17 |
| K_S | 1.15 | D_Sa_S_K | 1.25 |
| Sa_S | 1.67 | D_S_K_C | 0.97 |
|  |  | D_S_Sa_C | 1.82 |
|  |  | D_S_Sa_K | 1.48 |
|  |  | K_C_S_Sa | 1.19 |
|  |  | Sa_C_S_K | 2.26 |
|  |  | Sa_K_S_C | 2.19 |
| Average POL | **1.09** | **Average POL** | **1.57** |
| Standard deviation | **0.35** | **Standard deviation** | **0.46** |

*S: Smeekens; Sa: Saraiva; K: Klassert; C: Czakai; D: Dix

Table S2 – List of biomarker genes from each type of classifier

| Common Genes | Unique to single classifiers | Unique to combined classifiers |
| --- | --- | --- |
| RRAGD | ANTXR2 | ATP6V1D |
| RGCC | PPFIBP2 | BLVRA |
| IFNB1 | AGAP3 | TNFRSF14 |
| KLHL21 | RIN2 | RNF144B |
| TBC1D7 | GNPDA1 | ADA |
| ADGRE1 | PPIF | CXXC5 |
| TBC1D2 | BATF3 | CH25H |
| HMOX1 | SRXN1 | SDSL |
| HCAR2 | PHACTR1 | BCAR3 |
| PELI1 | TAGAP | TNFSF15 |
| DHRS9 | CCL8 | APOBEC3A |
| NCF1 | RGS1 | TNFSF10 |
| TNFSF14 | TRIM21 | DPYSL3 |
| CCR1 | CD86 | HK2 |
| TBC1D9 | SATB1 | NCF1C |
| SPRY2 | GLIPR2 | RTP4 |
| SOWAHC | UBASH3B | GLA |
| CEBPB | IVNS1ABP | ARHGEF3 |
| PLCXD1 | ANAPC4 | NSMAF |
| NCOA7 | TTC14 | KLF4 |
| CXCL11 | ACADVL | TMEM243 |
| NRIP3 | MGAT2 | TRMT5 |
| LGALS9 | CRYGS | CMTM7 |
| TNFSF13B | WDFY2 | GPAT3 |
| IL12B | CRIPAK | TGFBI |
| CEP135 | HACD3 | TMEM106A |
| IL27RA |  | ANKIB1 |
| ENC1 |  | UBA7 |
| ATP6V0A1 |  | FAM111A |
| PAPSS1 |  | SLC16A3 |
| ST3GAL5 |  | SPP1 |
| EVL |  | TLR7 |
| SCARB2 |  | EGR2 |
| SP140 |  | CHST12 |
| NOP16 |  | CALU |
| PCID2 |  | SLC7A7 |
| RBCK1 |  | EMP1 |
| SLFN12 |  | PRKAG2 |
| TRAFD1 |  | C14orf159 |
| STK26 |  | FXYD6 |
| HAVCR2 |  | RHOU |
| FAM46A |  | DDX60L |
| EDN1 |  |  |
| CLCF1 |  |  |
| CD40 |  |  |
| PARP4 |  |  |

Table S3 List of genes selected from the combined approach and their respective adjusted p-values (<0.05 was regarded to be significant)

| Gene symbol | Dix | Smeekens | Saraiva | Klassert | Czakai |
| --- | --- | --- | --- | --- | --- |
| ADA | 3.64E-06 | 1.12E-03 | 4.92E-01 | 1.89E-05 | 1.04E-03 |
| BCAR3 | 2.02E-06 | 2.51E-06 | 2.42E-02 | 5.47E-05 | 3.44E-03 |
| BLVRA | 3.89E-04 | 9.37E-03 | 6.99E-02 | 4.95E-02 | 6.14E-06 |
| CCR1 | 4.01E-03 | 4.95E-09 | 2.99E-01 | 1.37E-08 | 1.81E-02 |
| DHRS9 | 1.23E-05 | 1.79E-07 | 3.15E-01 | 3.74E-07 | 2.94E-03 |
| EDN1 | 2.57E-02 | 2.53E-20 | 1.21E-03 | 5.08E-01 | 1.73E-02 |
| EGR2 | 4.24E-05 | 2.30E-14 | 5.26E-01 | 1.49E-04 | 2.10E-04 |
| GLA | 2.40E-08 | 1.77E-11 | 1.80E-02 | 4.58E-07 | 1.46E-02 |
| HMOX1 | 7.92E-07 | 5.07E-04 | 3.50E-02 | 2.05E-06 | 2.15E-02 |
| PAPSS1 | 1.06E-02 | 1.82E-04 | 5.51E-01 | 9.31E-09 | 7.42E-04 |
| RNF144B | 5.56E-05 | 3.66E-03 | 5.05E-01 | 3.19E-08 | 3.14E-03 |
| RRAGD | 2.22E-08 | 1.74E-02 | 1.57E-01 | 2.86E-04 | 2.59E-04 |
| SCARB2 | 5.71E-01 | 4.62E-20 | 4.72E-02 | 2.82E-04 | 9.94E-04 |
| SDSL | 7.43E-05 | 2.00E-02 | 2.22E-01 | 5.97E-06 | 1.42E-02 |
| SOWAHC | 3.39E-06 | 2.04E-01 | 2.66E-02 | 3.70E-03 | 3.00E-03 |
| SPRY2 | 5.64E-08 | 1.66E-02 | 9.32E-03 | 3.21E-05 | 2.05E-03 |
| TBC1D7 | 1.88E-11 | 1.87E-03 | 1.31E-02 | 3.55E-03 | 7.82E-03 |
| TNFSF14 | 3.79E-11 | 4.07E-03 | 4.30E-02 | 2.18E-05 | 7.56E-03 |
| TNFSF15 | 1.16E-08 | 6.08E-02 | 9.32E-03 | 1.70E-05 | 4.15E-04 |

Table S4 Single Classifier Performances

|  | Smeekens | Saraiva | Klassert | Dix | Czakai | Average |
| --- | --- | --- | --- | --- | --- | --- |
| Sensitivity | 0.94 | 0.94 | 0.90 | 0.95 | 0.97 | 0.94 |
| Specificity | 0.98 | 0.97 | 0.99 | 0.96 | 0.99 | 0.98 |
| PPV | 0.98 | 0.97 | 0.99 | 0.95 | 0.99 | 0.98 |
| NPV | 0.94 | 0.94 | 0.92 | 0.95 | 0.97 | 0.94 |
| Accuracy | 0.96 | 0.95 | 0.95 | 0.95 | 0.98 | 0.96 |

Table S5 Combined Classifier Performances

|  | S_Sa | S_K | S_C | Sa_K | Sa_C | K_C | D_S | D_Sa | D_K | D_C | Average |
| --- | --- | --- | --- | --- | --- | --- | --- | --- | --- | --- | --- |
| Sensitivity | 0.97 | 0.95 | 0.95 | 0.95 | 0.98 | 0.97 | 0.95 | 0.97 | 0.94 | 0.97 | 0.96 |
| Specificity | 0.98 | 1.00 | 0.99 | 0.93 | 0.93 | 1.00 | 0.97 | 0.94 | 0.97 | 0.97 | 0.97 |
| PPV | 0.98 | 1.00 | 0.99 | 0.93 | 0.94 | 1.00 | 0.97 | 0.93 | 0.97 | 0.97 | 0.97 |
| NPV | 0.97 | 0.95 | 0.95 | 0.95 | 0.98 | 0.98 | 0.95 | 0.97 | 0.94 | 0.97 | 0.96 |
| Accuracy | 0.97 | 0.97 | 0.97 | 0.94 | 0.96 | 0.99 | 0.96 | 0.95 | 0.96 | 0.97 | 0.96 |

*S: Smeekens; Sa: Saraiva; K: Klassert; C: Czakai; D: Dix
